# Association of miR-34a Expression with Quality of Life of Glioblastoma Patients: A Prospective Study

**DOI:** 10.3390/cancers11030300

**Published:** 2019-03-04

**Authors:** Paulina Vaitkiene, Aiste Pranckeviciene, Rytis Stakaitis, Giedrius Steponaitis, Arimantas Tamasauskas, Adomas Bunevicius

**Affiliations:** 1 Laboratory of Molecular Neurooncology, Neuroscience Institute, Lithuanian University of Health Sciences, Eiveniu str. 4, LT-50161 Kaunas, Lithuania; rytis.stakaitis@lsmuni.lt (R.S.); giedrius.steponaitis@lsmuni.lt (G.S.); arimantas.tamasauskas@kaunoklinikos.lt (A.T.); 2 Institute of Biology Systems and Genetic Research, Lithuanian University of Health Sciences, Tilzes str. 18, LT-47181 Kaunas, Lithuania; 3 Laboratory of Behavioral Medicine, Neuroscience Institute, Lithuanian University of Health Sciences, Eiveniu str. 4, LT-50161 Kaunas, Lithuania; aiste.pranckeviciene@lsmuni.lt (A.P.); a.bunevicius@yahoo.com (A.B.)

**Keywords:** glioblastoma, health-related quality of life, miR-34a expression, depression, survival, prognosis

## Abstract

MiR-34a acts as tumor-suppressor by targeting many oncogenes related to proliferation, apoptosis, and invasion of gliomas. We studied the relationships between health-related quality of life (HRQOL), depression, and miR-34a expression status in patients with newly diagnosed glioblastoma (GBM). A comprehensive HRQOL assessment was completed by 38 patients with glioblastoma prior to surgical resection and included the European Organization for Research and Treatment of Cancer (EORTC) questionnaire for cancer patients (QLQ-C30) and the Brain Cancer-Specific Quality of Life Questionnaire (QLQ-BN20), the Patient Health Questionnaire-9 (PHQ-9), the Karnofsky performance index (KPS), and The Glasgow Outcome Scale (GOS). The miR-34a expression in glioblastoma tissue was measured using quantitative reverse transcription PCR. Our findings show that lower miR-34a expression is significantly associated with higher tumor volume, worse physical functioning, lower KPS, and greater depressive symptom severity of GBM patients. Moreover, analysis reveals that miR-34a effects might be gender specific, as stronger relationships between miR-34a and patient functioning measures were observed in males when compared to females. Despite the fact that, due to small sample size, our results should be considered as preliminary, our study suggests that miR-34a is associated with tumor burden and can be important for health-related quality of life, functional status, and mood symptoms of glioblastoma patients.

## 1. Introduction

Glioma is a rare and often devastating disease associated with significant functional impairment and short survival time [1,2]. Prediction of outcomes after brain tumor surgery is critical for treatment guidance and optimized use of healthcare resources. Currently, besides traditional outcome measures such as overall survival, progression-free survival, and radiological response to treatment, the value of patient-centered outcome measures is widely acknowledged [3]. Health-related quality of life (HRQOL), as an outcome measure, reflects the patient’s perspective on his or her disease, covering physical, psychological, and social aspects of patient’s functioning, as well as symptoms induced by the disease and/or its treatment [4]. Decreased HRQOL and depression in patients are sensitive predictors of shorter survival in glioma patients [3]. However, patient-centered outcome measures are rarely investigated in relation to biological biomarkers.

The need for glioma biomarkers with improved sensitivity and specificity has inspired research of small non-coding micro-RNAs (miRNAs). Previous studies report that MIR34A (miR-34a) can directly down-regulate several target mRNAs which encode proteins required for cell cycle transition (E2F3, MYCN, CCND1, c-MET, Notch1/Notch2), invasion and metastasis (Fra-1), mitogen-activated protein kinase pathways (MAP3K9), and anti-apoptotic function (Bcl-2) [5]. Expression profiling identifies miR-34a as one of the several microRNAs that are down-regulated in various types of cancer including neuroblastoma, leukemia, pancreatic and hepatocellular carcinomas, glioblastoma, breast, lung, and colon cancers [6]. On the contrary, other studies find that it functions as an oncogene promoting tumorigenesis in renal cell carcinoma, papillary thyroid carcinoma (PTC), and uterine cancers [7,8,9]. These studies across different types of cancers have contradictory results regarding miR-34a role in tumor progression.

Gender is an important factor that affects the risk of cancer occurrence and development, incidence, prognosis, and treatment response and sex-specific therapeutic strategies should be quite urgent in cancer treatment [10]. There is increasing evidence that miR-34a expression may be related to gender [10,11]. Sex and treatment-dependent regulation of miRNAs may explain the different treatment response of males and females. Therefore, it is important to examine the impact of miRNA expression in both sexes under different disease conditions. Although extensive studies explore the role of miR-34a in the glioblastoma cell lines [12], little is known about the relationship between the expression level of miR-34a in glioblastoma tissues and the quality of patient functioning. Therefore, in this study, we examine the associations between expression level of miR-34a in glioblastoma tissue and the spectrum of a patients’ presenting symptoms.

## 2. Results

### 2.1. Demographic and Clinical Characteristics Relationship with miR-34a Expression Levels

Social, demographic, and clinical characteristics of the sample are presented by miR-34a expression status in Table 1. Expression of miR-34a in tumor tissue was not related to any of demographic variables. The tendency that patients with lower miR-34a expression more frequently had frontal tumors can be observed and less of them were diagnosed with tumors located in more than one lobe of the brain. Patients with higher miR-34a expression were more frequently diagnosed with multifocal tumors, however none of these differences were statistically significant, most likely due to a small number of patients in the subgroups.

Patients with lower miR-34 expression had significantly greater tumor volume in contrast-enhanced T1-weighted sequences, when compared with patients with higher miR-34a expression (U = 34.0, *p* = 0.03), however no volume differences were found in T2 fluid-attenuated inversion recovery (FLAIR) image sequences (Table 1).

Additional analysis of tumor volume and miR-34a expression relationships in gender subgroups revealed stronger relationship between miR-34a expression and tumor volume in males when compared with females.

**Figure 1:**
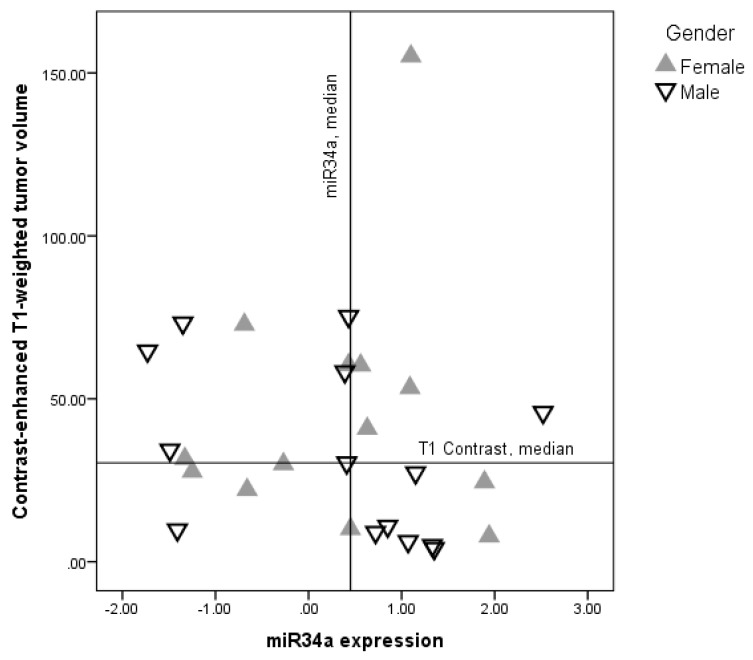
Relationship between miR-34a expression and contrast-enhanced T1-weighted glioblastoma tumor volume. Solid lines represent median values of either miR-34a expression (vertical) or T1 contrast median (horizontal). Gender dependent correlation between T1-weighted contrast-enhanced tumor volume and miR-34a expression was found in the male **▽** (Spearman rho = −0.53, *p* = 0.05) but not in the female ▲ (Spearman rho = −0.09, *p* = 0.78) subgroup.

In males, miR-34a expression correlated negatively with T1-weighted contrast-enhanced tumor volume (Spearman rho = −0.53, *p* = 0.05) (Figure 1). The correlation between miR-34a expression and tumor volume on FLAIR sequences was insignificant (Spearman rho = −0.25, *p* = 0.31). In females, there was no correlation between miR-34a expression and T1 contrast volume (Spearman rho = −0.09, *p* = 0.78), as well as no correlation between miR-34a expression and FLAIR tumor volume (Spearman rho = 0.18, *p* = 0.54).

### 2.2. Health Related Quality of Life and miR-34a Expression

Relationships between miR-34a expression levels and HRQOL indicators are shown in Table 2. Correlation analysis revealed a statistical tendency for higher miR-34a expression in tumor tissue to be positively related with physical functioning and total HRQOL reported by glioblastoma patients (Table 2). Correlation between miR-34a expression and physical functioning was stronger in males. Tendency for positive correlation between miR-34a expression and cognitive and social functioning was also observed in males, but not in females. Higher miR-34a expression was significantly negatively related to subjectively reported complaints in drowsiness. Similarly, the relationship between drowsiness and miR-34a expression was stronger in the male subsample.

### 2.3. Depression Correlation with miR-34a Expression

In addition to making criteria-based diagnoses of depressive disorders, the PHQ-9 is a reliable and valid measure of depression severity. The examination was conducted before the operation. Higher miR-34a expression was statistically significantly negatively correlated with depressive symptom severity, preoperatively (Table 2), both in the total sample of GBM patients and in males and females separately.

### 2.4. Functional Status

The Karnofsky Performance Scale (KPS) allows patients to be classified as to their functional impairment. This can be used to compare effectiveness of different therapies and to assess the prognosis in individual patients. Higher miR-34a expression in tumor tissue is significantly positively correlated with the KPS score on admission, indicating that patients with higher miR-34a expression have a better functional status before surgery. This association was slightly stronger in males when compared to females (Table 2). The miR-34a expression was not related to functional outcomes at discharge, assessed with the GOS.

### 2.5. Correlation of miR-34a Expression and Patient Survival

**Figure 2:**
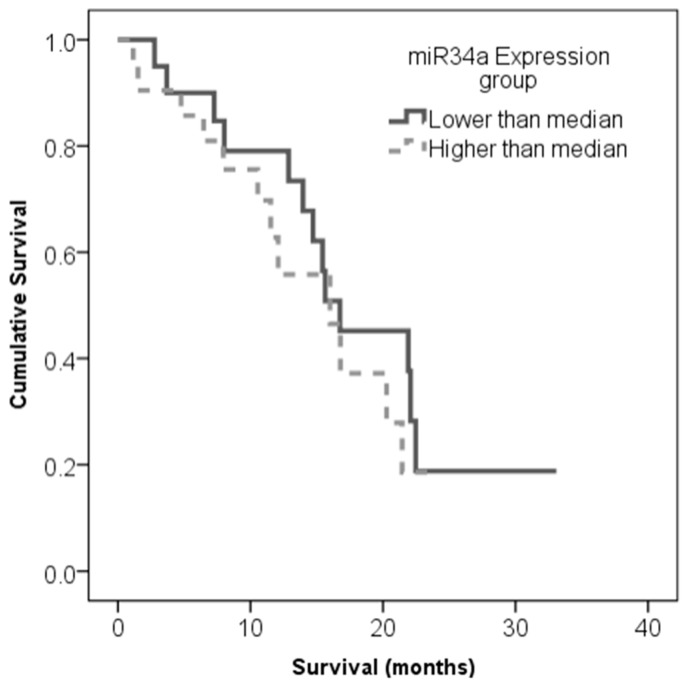
Kaplan–Meier survival curves in high and low miR-34a expression groups. No association between overall patient survival and miR-34a expression was found (Log-rank test, *χ*2 = 0.471, df = 1, *p* = 0.493).

The observed association of miR-34a expression with health-related quality of life and functional status indicated for us to check the association between patient survival and miR-34a expression. For this purpose, the miR-34a expression level values obtained from the complete set of 41 glioblastoma samples were divided into two categories as follows: Values that were lower than or equal to the median expression were ranked as “low” miR-34a expression levels and values that were higher than the median were ranked as “high” miR-34a expression levels. The Kaplan–Meier analysis using the log-rank test showed no association between overall patient survival and miR-34a expression (Log-rank test, χ2 = 0,471, df = 1, *p* = 0.493) (see Figure 2).

## 3. Discussion

This study, for the first time, reveals the relationships between HRQOL and miR-34a expression in patients with newly diagnosed glioblastomas. Previous studies indicate that miR-34a expression might be decreased in glioblastomas, as compared to lower grade gliomas and non-tumor brain tissue. Moreover, low levels of miR-34a were associated with a poor survival prognosis. However, more comprehensive studies are needed to confirm the significance of miR-34a expression levels for the glioblastoma patients [6,13,14,15].

Firstly, we analyzed the relationship between social, demographic, and clinical characteristics of glioblastoma patients and miR-34a expression levels in their tumor tissue and found that patients with lower miR-34a expression had significantly higher tumor volumes. These results are expected as previous studies show that miR-34a may act as tumor suppressor gene by targeting many oncogenes related to proliferation, differentiation, growth, apoptosis, and invasion [16]. The association of miR-34a expression with tumor size is also reported in other types of tumors, e.g. low miR-34a levels are associated with larger sizes of hepatocellular carcinoma and prostate cancer [17,18]. Meanwhile, Gao and colleagues did not notice any association between miR-34a expression and glioma tumor sizes [13]. Different results in the Gao et al. study may be attributed to a different study design, as gliomas of different grades are analyzed together. We analyze only glioblastoma patients, thus making our sample more homogeneous. Behaviour of low-grade gliomas is different than that of high-grade gliomas, thus different relationship between tumor volume and miR-34a expression might be expected as a function of tumor grade. Gao and colleagues also evaluate tumor size by using tumor diameter while we employ volumetric analysis [13]. More detailed research in larger patient samples is needed to confirm these results.

Some gender differences in GBM risk and the course of the illness were recently reported. GBM incidence rates in males are slightly higher when compared to females, however males have some survival advantages over females during the first year after diagnosis but with no difference thereafter [19,20]. Gender might be important for treatment-dependent regulation of miRNA expression and may explain the differential treatment response of males and females [10]. Thus, it is important to examine the impact of miRNA-expression in both sexes individually and under different disease conditions. There is increasing evidence that the expression of miR-34a may be related to gender and in response to therapy. For example, miR-34a was up-regulated in prostate adenocarcinoma, male group, and was not abnormally expressed in the other related cancer groups [10]. There are sex differences in response to miRNA-34a therapy in mouse models of cardiac disease [11]. Thus, it is important to examine the impact of miRNA expression in both sexes individually. In our study, additional analysis of tumor volume and miR-34a expression relationships in gender subgroups reveals stronger relationship between miR-34a expression and tumor volume in males, when compared to females. Although the exact mechanism of miR-34a regulation across genders still needs to be discovered, preliminary findings indicate that various hormone factors might participate in miR-34a expression regulation. For example, the thyroid hormone 3,3,5-triiodo-L-thyronine (T3) is shown to induce the expression of miR-34a [21] and reduced T3 levels are linked to worse HRQOL and shorter survival of brain tumor patients, including those with gliomas [22,23]. Additional hormones to be considered in miR-34a regulation are estradiol (E2), as shown in human breast cancer [24]. However, in our sample of glioblastomas, the difference in expression between miR-34a and gender has not been established. Nevertheless, with increasing evidence that miR-34a expression can be controlled by the hormone, we have decided to explore the differences of HRQOL, functional status, or depression linking to miR-34a expression in more detail, not only across all the glioblastoma samples but also between genders.

Worse perceived HRQOL is shown to predict shorter survival of glioma patients [25]. Identification of molecular markers, which could act as predictors of patients’ health status, is important in order to develop novel therapeutic strategies aiming to improve prognosis and to optimize the health status of glioblastoma patients. There is a tendency for an association between higher miR-34a expression and better physical functioning and overall HRQOL. The correlation between higher miR-34a expression and better physical functioning was stronger in males. The tendency for a positive correlation between miR-34a expression and cognitive and social functioning is also observed in males, but not in females. Patients with a higher miR-34a expression also scored higher on the KPS before surgery. However, miR34a expression was not significantly related to short term patients’ functional outcomes at the time of discharge.

Given the poor prognosis of glioblastoma, depression stands to worsen outcomes when it develops concomitantly [26]. Despite this common interaction, relatively little research has been performed on the development of depression associated with glioblastoma. One reason for this is that the pathophysiological development of depression and glioblastoma share several pathways, including altered regulation of the 5-HT receptor, norepinephrine, and 3′:5′-cyclic monophosphate [26]. We find that patients with a lower expression of miR-34a reported more severe depressive symptoms. These results are in line with Azavedo et al. [27], who report an association between miR-34a expression in postmortem brain tissue of patients with Major Depressive Disorder and Bipolar Disorder. In animal models, miR-34 family is related to stress and anxiety response [28]. Current evidence suggests that the miR-34 family might have a critical function in regulating the behavioral and neurochemical response to acute stress and in inducing stress-related amygdala neuroplasticity [29]. However, studies with many more cases will be needed to carefully elucidate the better awareness of depression when it occurs in conjunction with miR-34a expression and to encourage optimal patient care and future research to identify potential molecular pathways between them.

Previous studies provide contradictory results on the importance of miR-34a expression for the survival of glioma patients. Gao and colleagues find that grade III glioma and glioblastomas with lower miR-34a expressions correlates with worse progression-free patient survival and overall survival [10]. Meanwhile, Toraih and colleagues do not find any significant associations between miR-34a expression levels and overall survival of glioblastoma patients [6]. In contrast to these previous studies, Genovese and colleagues, in two independent cohorts of glioblastoma, show that glioblastomas with low-expressing miR-34a have better outcomes, with longer survival overall [15]. In our sample of glioblastoma patients, no statistically significant associations between miR-34a expression and overall survival are found. Further studies are needed to confirm miR-34a expression significance in glioblastomas.

Several limitations of the current study should be acknowledged. A relatively small sample size limited the statistical power of our analysis and prevented us from employing more sophisticated and multivariate statistics. Preoperative MRI images were collected retrospectively and they were available for only 61% of total sample. It might be expected that the size of the tumor is a significant covariate linking miR-34a expression with various aspects of patients functioning, thus, further studies investigating miR-34a in the context of clinical factors are needed. However, this study presents one of the first attempts to link molecular tumor data with patients functioning, assessed by patients themselves as well as their doctors. Patient assessments were performed prospectively and provide us with interesting relationships between micro and macro levels of patient functioning.

## 4. Materials and Methods

### 4.1. Procedures

The study protocol and consent procedures were approved by the Ethics Committee for Biomedical Research of the Lithuanian University of Health Sciences (LUHS) (P2-9/2003 and BE-2-3). Written informed consent was obtained from each study patient before inclusion in the study.

Consecutive adult patients admitted for surgery for suspected glioblastoma based on brain MRI, at the Department of Neurosurgery, Hospital of LUHS, Kaunas, Lithuania in a period from October 2015 until May 2017, were invited to participate in this prospective observational cohort study. The study exclusion criteria included severe cognitive deficits and/or neurological impairment leading to inability to complete all study tasks. Neuropsychological assessment was performed, from two to three days before brain tumor surgery, by a certified medical psychologist. The medical history, clinical characteristics, and functional status of the study patients were recorded by the study neurosurgeon. Histological brain tumor diagnoses were verified from postoperative pathology reports. Pre-operative MRI images were obtained from medical documentation.

### 4.2. Samples

Forty-six patients with histologically confirmed glioblastoma participated in the study. Data of five patients was excluded due to failed miRNA analysis. Thirty-eight (92.7%) patients completed health related quality of life (HRQOL) and depression questionnaires. Functional status was assessed in 37 (90.2%) patients. Preoperative MRI data was available for 25 (61.0%) patients.

### 4.3. Questionnaires

The European Organization for Research and Treatment of Cancer Quality of Life Questionnaire QLQ-30 [30] and QLQ-BN20 questionnaires [31,32] were used to evaluate preoperative health related quality of life (HRQOL) and brain tumor related symptoms. Both questionnaires were previously validated for HRQOL assessment in Lithuanian brain tumor patients [33].

The QLQ-C30 contains 30 items that were designed to assess global health status, functional status, role functioning, emotional functioning, cognitive functioning, social functioning, and various cancer related symptoms. Raw scores were linearly transformed to 0–100 scales with higher scores indicating better global health, functional status, and greater general HRQOL.

The QLQ-BN20 is a 20-item self-rating instrument that was designed as the QLQ-C30 supplement for evaluation of HRQOL specifically in brain tumor patients. It addresses future uncertainty, visual disorder, motor dysfunction, communication deficits, and other common BT-related symptoms. The QLQ-BN20 scores were linearly transformed to a 0–100 scale with higher score indicating greater BT-related symptom severity.

The Patient Health Questionnaire-9 (PHQ-9) [34] is a brief self-report tool for screening, diagnosing, monitoring, and measuring the severity of depression. The PHQ-9 is based on the Diagnostic Statistical Manual-IV depression diagnostic criteria and it is recommended for depression screening in glioma patients [35,36].

The Karnofsky performance scale (KPS) [37], was used for assessment of functional status. The KPS is an 11-point rating scale that is designed to measure a patient’s ability to carry his/her normal activities and dependence on help and nursing care. The total KPS score ranges from 100 (normal functioning) to 0 (death).

Functional outcomes at hospital discharge were evaluated by a neurosurgeon using The Glasgow Outcome Scale (GOS) [38]. The GOS ranges from 1 (death) to 5 (good recovery) and it is widely used for research purposes in neurosurgical patients.

### 4.4. Tumour Volume Measurements

Tumor segmentation and volume measurements were performed using the 3D Slicer medical image computing platform, version 4.3.1 (www.slicer.org) [39]. We used the most recent pre-operative structural MRI imaging data, which was performed on 1.5T or 3T MRI scanners. For the purpose of this study, we used T1-weighted contrast enhanced and T2-FLAIR weighted sequences. T1-contrast enhancing tumor volume (in cm^3^), representing tumor necrotic core, and T2-FLAIR hyper-intense tumor volume, representing tumor infiltrations/edema, were calculated. All volumetric analyses were performed by a trained neurosurgeon. The rater was blinded to microRNA and psychological assessment data.

### 4.5. Small RNA Extraction, Micro RNA cDNA Synthesis and qPCR Performance

Small RNA (<200 nt) was extracted from snap-frozen (−196 °C) post-surgical tumor samples applying cryogenic mechanical grinding, ultrasonic homogenization, and using a “mirVana™ miRNA Isolation Kit” (Catalog nr: AM1560). Quality and quantity of extracted small RNAs were evaluated with Agilent “2100 Bioanalyzer” (Part nr: G2939BA) and “Small RNA analysis kit” (Part nr: 5067-1548). A measure of 10 ng of purified micro RNA was synthesized to cDNA using a “TaqMan™ Advanced miRNA cDNA Synthesis Kit” (Catalog nr: A28007) and the expression profile of mature micro RNA 34a (hsa-miR-34a-5p) was detected by performing quantitative RT-PCR (qPCR) on “Applied Biosystems 7500 Fast Real-Time PCR System” in 3 replicates using “TaqMan™ Fast Advanced Master Mix” (Catalog nr: 4444557) in addition to hsa-miR-191-5p, hsa-miR-361-5p (as referenced), and hsa-miR-34a-5p probes from “TaqMan™ miRNA Advanced Assay” product line (Assays ID: 477952_mir, 478056_mir, and 478048_mir, respectively). Fluorescent data were converted to cycle threshold (Ct) measurements and relative quantitation of hsa-miR-34a-5p was calculated according to the following formulas:

1) $\Delta Ct_{miRNA}=MeanCt_{miRNA}-\sqrt{MeanCt_{miR-191}\times MeanCt_{miR-361}}$
2) $2^{-\Delta Ct_{miRNA}}$

In order to quantify samples in 95% of the cases, samples with a standard deviation of more than 0.25 were eliminated from the analysis.

### 4.6. Statistical Analysis

The SPSS Statistics 19 (SPSS Inc., Chicago, IL, USA) software package was used for statistical analysis. Chi-square and Mann–Whitney tests were used to evaluate associations among miR-34a expression levels and clinical parameters. The relationship between patients’ functioning and miR-34a expression was evaluated using Spearman correlation analysis. A Kruskal–Wallis test was used to reveal the difference across medians of miR-34a expression. The significance level was defined as a *p* value less than 0.05. The Kaplan–Meier method was used to estimate survival functions. For comparing survival time distribution between groups, the log-rank test was used.

## 5. Conclusions

Taken together, the findings of our study suggest that some molecular markers might be important for health-related quality of life, functional status, and depressive symptoms of glioblastoma patients. That is, the slower proliferation rate of tumors with higher miR-34a expression may allow for greater neuroplasticity by offering the brain more time for reorganization in response to invading tumors. Due to the small sample size, our results should be considered as preliminary. Thus, further studies in miR-34a expression in glioblastoma patients, addressing possible gender differences, are strongly encouraged.

## Figures and Tables

**Table 1:** Social, demographic and clinical characteristics in total study sample and miR-34 subgroups.

Characteristics	Total Sample	Lower than Median miR34 Expression	Equal or Higher than Median miR34 Expression
N (%)			
Gender Females Males	23 (56.1%) 18 (43.9%)	13 (56.5%) 7 (38.9%)	10 (43.5%) 11 (61.1%)
Marital status Living alone With partner	6 (14.6%) 35 (85.4%)	3 (50.0%) 17 (48.6%)	3 (50.0%) 18 (51.4%)
Education Lower than university University degree	18 (43.9%) 23 (56.1%)	8 (44.4%) 12 (52.2%)	10 (55.6%) 11 (47.8%)
Tumor location Frontal Temporal Parietal Occipital Two or three lobes	14 (34.1%) 8 (19.5%) 6 (14.6%) - 13 (31.7%)	10 (71.4%) 3 (37.5%) 3 (50.0%) - 4 (30.8%)	4 (28.6%) 5 (62.5%) 3 (50.0%) - 9 (69.2%)
Tumor side Right Left Bilateral	19 (46.3%) 19 (46.3%) 3 (7.3%)	10 (52.6%) 9 (47.4%) 1 (33.3%)	9 (47.4%) 10 (52.6%) 2 (66.7%)
Lesion Solitary Multifocal	34 (82.9%) 7 (17.1%)	19 (55.9%) 1 (14.3%)	15 (44.1%) 6 (85.7%)
Median			
Volume T1 Contrast enhanced T2 FLAIR weighted	31.0 116.7	58.3 144.3	24.4* 114.2

* U = 34.0, *p* = 0.03.

**Table 2:** Relationship between health-related quality of life indicators, clinical evaluation of patient’s functioning, and miR-34 expression in glioblastoma patients. Spearman rho.

Scales and Domains	miR-34 Expression					
	Total Sample		Females		Males	
	rho	Sig.	rho	Sig.	rho	Sig.
	**Health-related Quality of life**					
**EORTC QLQ-C30 ^A^**						
Global evaluation of health	−0.05	0.76	−0.11	0.62	0.05	0.86
Physical functioning	**0.30**	**0.06**	0.18	0.40	**0.66**	**0.01**
Role functioning	0.05	0.78	−0.13	0.56	0.27	0.32
Emotional functioning	0.14	0.39	0.15	0.49	0.27	0.32
Cognitive functioning	0.11	0.52	−0.09	0.68	**0.44**	**0.09**
Social functioning	0.26	0.12	0.09	0.67	**0.44**	**0.09**
QLQ C30 Total Score	**0.31**	**0.06**	0.26	0.25	0.24	0.36
**EORTC QLQ-BN20 ^B^**						
Future uncertainty	−0.11	0.50	−0.14	0.52	−0.07	0.81
Visual difficulties	0.06	0.74	0.25	0.25	−0.22	0.43
Communication	0.15	0.38	0.18	0.42	−0.14	0.62
Motor difficulties	0.13	0.42	0.23	0.30	−0.17	0.52
Headaches	−0.08	0.64	0.07	0.70	−0.19	0.49
Seizures	0.15	0.36	−0.16	0.46	0.42	0.12
Drowsiness	**−0.34**	**0.03**	−0.20	0.37	**−0.49**	**0.05**
Hair loss	−0.01	0.97	0.33	0.13	−0.42	0.11
Itchy skin	−0.06	0.71	0.13	0.54	−0.27	0.32
Leg weakness	−0.26	0.11	−0.17	0.45	−0.42	−0.11
Bladder control	0.19	0.24	**0.37**	**0.08**	−0.01	0.98
	**Depression**					
**PHQ-9 ^C^**	**−0.36**	**0.03**	**−0.36**	**0.09**	**−0.37**	0.16
	**Level of functioning**					
**KPS at time of admission ^D^**	**0.36**	**0.03**	0.19	0.41	0.34	0.22
**GOS at time of discharge ^E^**	0.17	0.30	0.11	0.62	0.09	0.74

^A^ The European Organization for Research and Treatment of Cancer Quality of Life Questionnaire QLQ-30. Higher scores represent better functioning; ^B^ The European Organization for Research and Treatment of Cancer Quality of Life Questionnaire, Brain tumor module QLQ-BN20. Higher scores represent higher symptom burden. ^C^ Patient Health Questionnaire-9. Higher scores indicate higher levels of depression. ^D^ Karnofsky Performance Scale. Higher scores represent better functioning.^E^ Glasgow Outcome Scale. Bolded values indicate significant associations. Higher scores represent better functional outcomes after surgical treatment.
